# Effect of Anti-ApoA-I Antibody-Coating of Stents on Neointima Formation in a Rabbit Balloon-Injury Model

**DOI:** 10.1371/journal.pone.0122836

**Published:** 2015-03-30

**Authors:** Aart C. Strang, Menno L. W. Knetsch, Leo H. Koole, Robbert J. de Winter, Allard C. van der Wal, Carlie J. M. de Vries, Paul P. Tak, Radjesh J. Bisoendial, Erik S. G. Stroes, Joris I. Rotmans

**Affiliations:** 1 Department of Vascular Medicine, Academic Medical Center, Amsterdam, The Netherlands; 2 Department of Biomedical Engineering/Biomaterials Science, Maastricht University, Maastricht, The Netherlands; 3 Department of Cardiology, Academic Medical Center, Amsterdam, The Netherlands; 4 Department of Pathology, Academic Medical Center, Amsterdam, The Netherlands; 5 Department of Biochemistry, Academic Medical Center, Amsterdam, The Netherlands; 6 Department of Clinical Immunology and Rheumatology, Academic Medical Center, Amsterdam, The Netherlands; 7 Heart Research Institute, Newtown, NSW 2042, Australia; and Centenary Institute, Newtown, NSW, 2042, Australia; 8 Department of Nephrology, Leiden University Medical Center, Leiden, The Netherlands; University of Milan, ITALY

## Abstract

**Background and Aims:**

Since high-density lipoprotein (HDL) has pro-endothelial and anti-thrombotic effects, a HDL recruiting stent may prevent restenosis. In the present study we address the functional characteristics of an apolipoprotein A-I (ApoA-I) antibody coating *in vitro*. Subsequently, we tested its biological performance applied on stents *in vivo* in rabbits.

**Materials and Methods:**

The impact of anti ApoA-I- versus apoB-antibody coated stainless steel discs were evaluated *in vitro* for endothelial cell adhesion, thrombin generation and platelet adhesion. *In vivo*, response to injury in the iliac artery of New Zealand white rabbits was used as read out comparing apoA-I-coated versus bare metal stents.

**Results:**

ApoA-I antibody coated metal discs showed increased endothelial cell adhesion and proliferation and decreased thrombin generation and platelet adhesion, compared to control discs. In vivo, no difference was observed between ApoA-I and BMS stents in lumen stenosis (23.3±13.8% versus 23.3±11.3%, *p*=0.77) or intima surface area (0.81±0.62 mm^2^ vs 0.84±0.55 mm^2^, *p*=0.85). Immunohistochemistry also revealed no differences in cell proliferation, fibrin deposition, inflammation and endothelialization.

**Conclusion:**

ApoA-I antibody coating has potent pro-endothelial and anti-thrombotic effects *in vitro*, but failed to enhance stent performance in a balloon injury rabbit model *in vivo*.

## Introduction

The introduction of stents has increased the success of percutaneous coronary interventions (PCI) by reducing coronary artery restenosis rates.[1] This advantage, however, comes at a price. Bare metal stents (BMS) are more prone to in-stent restenosis (ISR) and their pro-thrombotic capacity may yield occlusion rates of up to 24% in the absence of pharmacological treatment.[2] Drug-eluting stents (DES) have proven highly effective through local delivery of antiproliferative drugs,[1,3] albeit the imminent threat of late stent thrombosis (LST) has hampered their success. Thus, a comparative meta-analysis revealed that long-term cardiac death rates for BMS and DES were not significantly different.[4] These results highlight the need for novel stents with better long-term performance compared to both BMS and DES.

We hypothesized that stent performance could be enhanced by coating the stent struts with anti-apolipoprotein A-I (ApoA-I) antibodies, aimed to attract high-density lipoprotein (HDL) to the stent. Indeed, HDL has been shown to carry a wide array of anti-inflammatory and anti-proliferative proteins, [5] which collectively have the capacity to prevent intimal hyperplasia. [6–8] In addition, HDL has also been shown to promote recruitment of endothelial progenitor cells, thereby promoting restoration of an intact endothelial monolayer covering the stent struts as well as the injured artery wall.[9] The physiological role of the endothelium is expected to reduce the inflammatory response and decrease coagulation activation with subsequent thrombus formation.[10,11]

In the present study, we evaluated the efficacy and therapeutic potential of anti-ApoA-I antibody coating on metal discs *in vitro* and bare metal stents *in vivo*. First, we addressed whether anti-ApoA-I coating improved the vascular homeostasis by improving endothelial cell adhesion, proliferation and decreasing thrombogenicity *in vitro*, compared to a bare-metal surface. Subsequently, we compared neointimal hyperplasia in a balloon-injury model in rabbits *in vivo* using an anti-ApoA-I coated versus bare-metal stent.

## Methods

### In vitro studies

The anti–human monoclonal ApoA-I antibody, ApoB100 antibody and isotype control IgG antibody were covalently coupled to stainless steel discs (5 mm diameter; double-sided). The surfaces with immobilized ApoA-I antibody (Clone 2F1, Ottawa Heart Institute Research Corporation, Ottawa, Canada)[12] were treated with human HDL (Sigma-Aldrich, Zwijndrecht, The Netherlands) or oxidized (ox)-HDL (0.2 mg/ml). Oxidized lipoproteins were obtained by dialysis of 0.8 mg/ml solutions of HDL or LDL against 5 μM Cu SO4 for 20 hours and using Slide-A-Lyzer with MWCO of 3,500 (Thermo Fisher, Etten-Leur, The Netherlands).[13] The surfaces with ApoB antibody (Clone 1D1, Ottawa Heart Institute Research Corporation) were incubated with human LDL (Sigma-Aldrich, Zwijndrecht, The Netherlands) or ox-LDL (0.2 mg/ml), while the surfaces with the isotype control IgG antibody were treated with a mixture of HDL and LDL or ox-HDL and ox-LDL (0.2 mg/ml). Ox-HDL, LDL and ox-LDL were used as negative control.

Human microvascular endothelial cells (HMEC-1; obtained from The Breakthrough Breast Cancer Research Center, London, England) were grown in MDCB-131 medium supplemented with 10% FBS, 2 mM L-glutamine, 1 μg/ml hydrocortisone, 10ng/ml recombinant h-EGF, and antibiotics (100U/ml penicillin, 100 μg/ml streptomycin, 0.25 μg/ml amphothericin B).[14] In order to determine proliferation of HMEC-1 on the different surfaces, metal discs were incubated for 1 hour with HDL, LDL, or a 1: 1 mixture of both. After washing, HMEC-1 cells were deposited on the discs and allowed to adhere for 1 hour at 37 ºC. After addition of medium, the discs were incubated for 1, 2 or 4 days. Subsequently, the discs were rinsed and frozen at -80ºC. The number of adhered cells to the metal surfaces was determined using the CyQuant kit (Life Technologies, Breda, The Netherlands).[15]

In order to quantify HMEC-1 adhesion, pre-incubated metal discs were put in a sterile 2.0 ml tube and incubated with 1.5x10^5^ cells in 0.8 ml MDCB-131 medium for 20 hours at 37 ºC under rotation. After rinsing, discs were stored at -80ºC. The number of adhered cells was determined using the CyQuant kit. Thrombin generation was determined in a static set-up, [16] (described in detail in S1 Text.

Platelet adhesion was determined using PRP that was prepared as described above. Metal discs were pre-incubated with HDL, LDL, or a HDL/LDL mixture and incubated with PRP for 1 hour at 37 ºC under continuous stirring at 150 rpm. Subsequently, the discs were washed with phosphate buffered saline (PBS), and the number of adhered platelets was determined using the CytoTox kit (Promega, Leiden, The Netherlands).[17] The modified surfaces were incubated with native or oxidized versions of HDL or LDL and treated with PRP under identical conditions as described above. Oxidized HDL and LDL were used to rule out the effect of oxidative modification on platelet activation.[18] Platelet activation was studied by fixing platelets adhered to modified surfaces with cold 2.5% glutaraldehyde in PBS. After careful washing with PBS, the samples were dehydrated with an ethanol series followed by incubation in hexamethyldisilazane (Aldrich, Zwijndrecht, The Netherlands) in order to achieve rapid drying.[19] Subsequently the samples were sputter coated with gold and observed using a SEM (Philips XL30 Scanning Electron Microscope, Philips, Eindhoven, The Netherlands). Photographs of randomly chosen areas were taken, and the morphology of the platelets was recorded according to the method described by Cooper *et al*.[20]

#### Stent coating

The anti–human monoclonal ApoA-I antibody was covalently coupled to struts of a 9mm x 3mm R-stent evolution 2 (OrbusNeich, US) at highest surface density (100%) using a proprietary multistep process (Ssens, The Netherlands). The R-stent has 316L stainless steel struts with dimensions of 0.09mm x 0.10mm, which are smooth and electropolished, and achieve 17% vessel wall coverage when implanted in the artery. The stent has an open, double helical design with recoil of less than 4% after implantation. The ApoA-I antibody coating was similar to the 100% density ApoA-I coated disks. An untreated R-stent was used as BMS comparator.

### In vivo study

#### Rabbit model

The study protocol was reviewed and approved by the Institutional Animal Care and Research Committee at the Academic Medical Center, Amsterdam, The Netherlands (protocol number DCA101095) and conforms to the Directive 2010/63/EU of the European Parliament. We used 15 female New Zealand White rabbits (3.0–3.5kg), which were treated with acetylsalicylic acid 38mg/day started 5 days prior to stent implantation. The rabbit model of balloon-induced artery injury is an established model to test intravascular stent devices since it is characterized by fast development of intima hyperplasia (described in detail in S2 Text).[21–23] In short, after induction of anaesthesia, stents were placed via cannulation of the left carotid artery, followed by endothelial denudation by pulling an inflated balloon through the iliac arteries. Directly thereafter, ApoA-I coated versus BMS stents were pair wise implanted in both endothelial denudated iliac arteries. During the experiment, rabbits had free access to food (AB-diets, high fibre complete diet).

At 28 days after stenting, the rabbits were anesthetized. Via the abdominal cavity, the retroperitoneal infrarenal aorta was cannulated with an 18G cannula and after intravenous injection of heparin 200IU/kg, angiography of the iliac arteries was performed. Subsequently, the animals were euthanized and the iliac arteries were flushed with saline, followed by perfusion-fixation using Neutral Buffered Formalin (NBF). The stented arteries were taken over a distance from the aorta-iliac bifurcation to one cm distal of the stent, fixated in NBF for 24 hours and subjected to the tissue embedding procedure.

After dehydration, the harvested arteries were stepwise embedded in MMA/BMA mix in a 1:1 ratio and allowed to harden for 24 hours under a vacuum at 4ºC. Using a microtome with diamond knife, 10 μm thick transversal sections were cut from 3 regions (proximal, middle and distal) of the stented arteries and 2 peri-stent regions proximal and distal to the stented artery. The middle region (region 3) is defined as 30 sections taken from the exact centre of the stent. The proximal and distal regions (regions 2 and 4) are defined as the 30 sections cut at a distance from 100μm from the stent edge, inside the stent, inwards. The 2 peri-stent regions (regions 1 and 5) are defined as the 30 sections cut at a distance from 100μm from the stent edge, outside the stent, outwards. Sections were stretched on a 60ºC bath of 40% acetone and adhered to glass slides using 70% Ethanol overnight at 60ºC under mechanical pressure.

Three randomly selected sections of any of the 5 regions were used for staining of the arterial laminae. Sections were stained using Haematoxilin-eosin staining, Lawson staining and immunohistochemical stainings detecting α-smooth muscle actin (α-SMA), Ki-67, rabbit macrophage-specific monoclonal antibody, von Willebrand factor and fibrin. Details about these stainings and morphometrical analysis are described in detail in S3 and S4 Texts, respectively.

Immunohistochemical analysis was performed by 2 separate reviewers, who were both blinded for type of stent coating. The mean score per stent was based on the average score of 2 randomly selected slides taken from both the proximal and distal stented area. Cell proliferation, inflammation, fibrin deposition and endothelialisation of the stented artery were scored (described in detail in S5 Text).[24–27] One rabbit was sacrificed after 28 days to perform scanning electron microscopy (SEM) on the implanted stents. For this purpose, the stented segment of the iliac artery was fixed in 2% glutaraldehyde and processed for SEM imaging, described in detail in S6 Text).

### Statistical analysis

Statistics were performed using IBM SPSS statistic (version 19) and graphs were constructed using Prism Graphpad (version 5). Based on previous studies utilizing this bilateral vascular injury in rabbits for a side-to-side comparison of two different stent modalities, we determined that for a difference of 25% in lumen stenosis with a standard deviation of 30% within one animal the minimum number of rabbits needed was 12 (power of 0,80 and a type I error probability of <0,05). Data are expressed as mean **±** SD. Significance of differences in *in vitro* tests was tested using one-way ANOVA. Comparisons of histological findings between BMS-stent and ApoA-I-coated stent were made by the Wilcoxon signed ranks test. Comparisons of immunohistochemistry results were made by Wilcoxon signed ranks test. A probability value of *P* < 0.05 was considered significant.

## Results

### In vitro studies

#### HMEC-1 cell growth and adhesion

**Fig 1:**
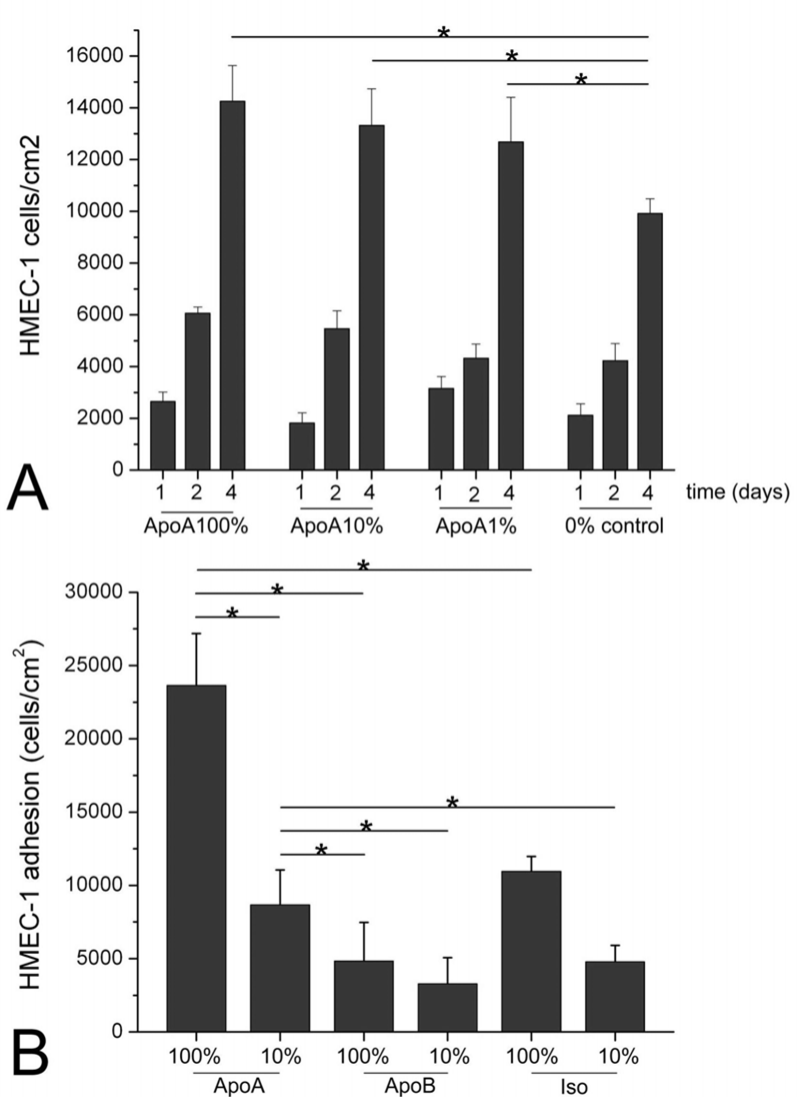
HMEC-1 cell growth on coated and uncoated disks. HMEC-1 cell growth on ApoA-I-coated discs in 100%, 10% and 1% density, incubated in HDL after 1, 2 and 4 days, compared to similarly incubated uncoated discs (control). Error bars indicate Standard Deviation (SD) of mean (n = 3). (* = *P*<0.05). HMEC-1 cell adhesion (B) on disks—HMEC-1 cell adhesion on ApoA-I-, ApoB- or Isotype-coated discs with 100% and 10% antibody density, incubated for 4 days. Error bars indicate Standard Deviation (SD) of mean (n = 4). (* = *P*<0.05)

After 4 days of incubation, the number of HMEC-1 cells on the anti-ApoA-I antibody coated surfaces was significantly higher compared to the isotype-antibody control, independent from antibody concentration (10% and 100%) (Fig 1A; p<0.05). There was an increased proliferation of HMEC-1 cells after 4 days on the surfaces with the highest density of anti-ApoA-I antibody, compared to those with lower densities (p<0.05).

The anti-ApoA-I antibody coated surface with the highest antibody density was associated with a higher HMEC-1 adhesion under dynamic conditions as compared to the isotype controls (p<0.05; Fig 1B). No effect on HMEC-1 adhesion was observed for the surfaces with the lowest anti-ApoA-I and anti-ApoB antibody density.

#### Thrombin generation

**Fig 2:**
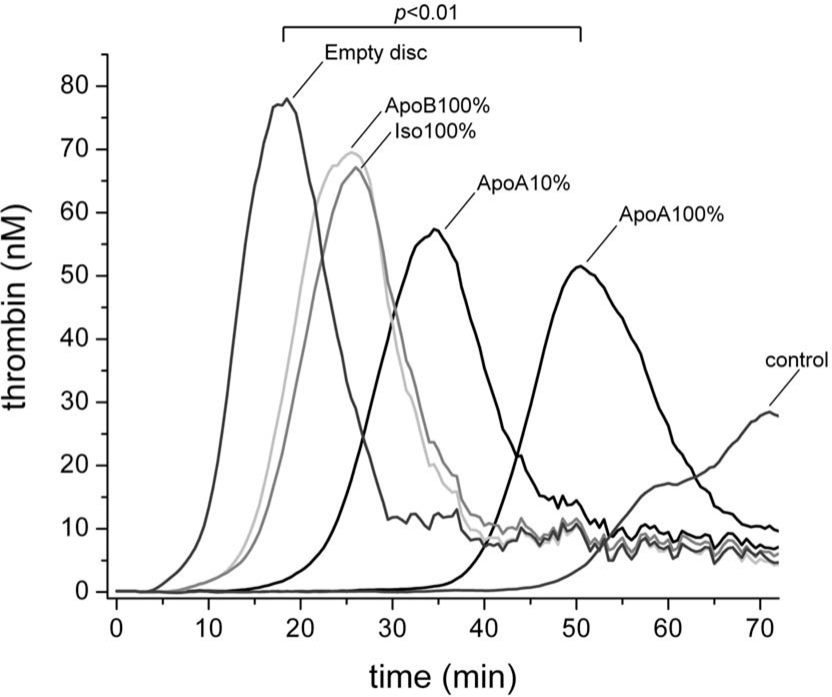
Thrombin generation on coated discs. Thrombin generation for discs coated with either ApoA-I-, ApoB-, Isotype- or no antibody (empty discs), compared to thrombin generation in absence of a disc (negative control). (n = 4).

Fig 2 shows that the anti-ApoA-I antibody coated surfaces that were incubated with HMEC-1 caused a significant prolongation in the thrombin generation time when compared to uncoated surfaces (*p*<0.01). In addition, lowered peak thrombin and the total amount of thrombin produced (*p*<0.05) was observed as compared to the isotype control. These anticoagulant effects increased with a higher density of the anti-ApoA-I antibody. In contrast, anti-ApoB antibody covered discs had no impact on the thrombin generation.

#### Platelet adhesion and activation

**Fig 3:**
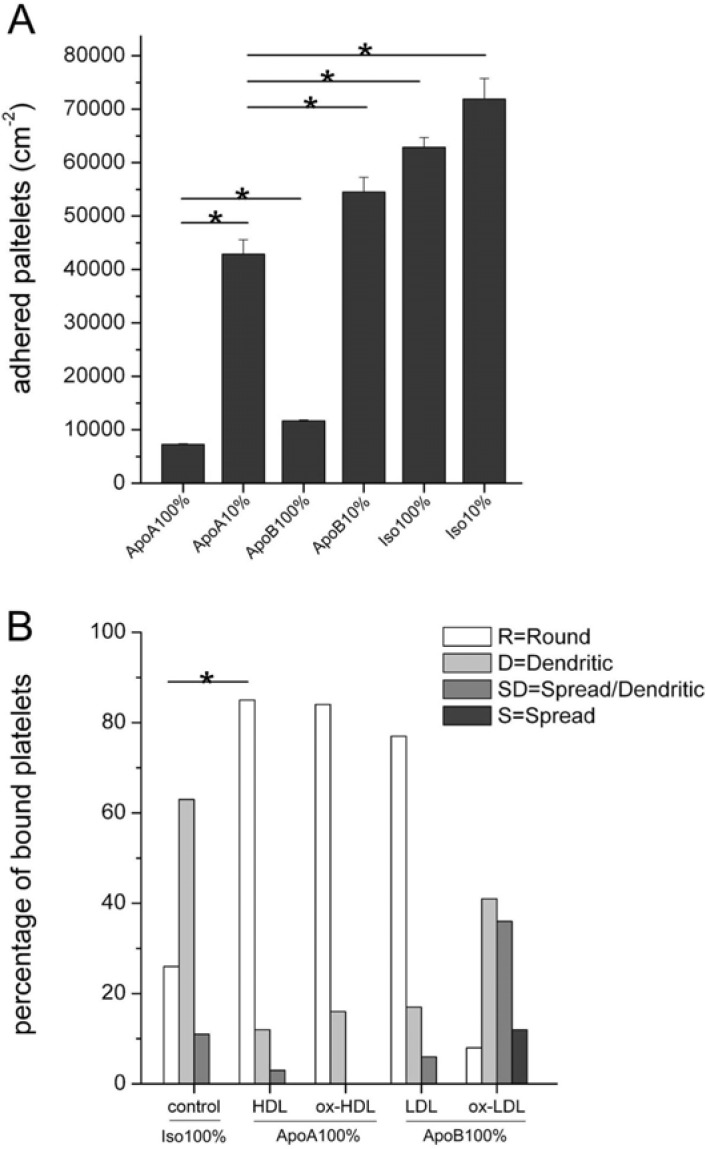
**A. Platelet adhesion on coated disks**. Platelet adhesion: SEM analysis of adhered platelets on surfaces coated with ApoA-I-, ApoB- or isotype antibody after corresponding pre-treatment. The number of adhered platelets are given as the mean ± SD (n = 3). (* = *P*<0.05). **B. Platelet morphology**. The morphology of the adhered platelets was divided into 4 different classes representing various degrees of activation, ranging from “round” (weak), “dendritic” (intermediate), “spread/dendritic” (strong) to “spread” (very strong). The data are presented as the percentage of platelets exhibiting the indicated morphology. Data error bars are the standard deviation of the mean (n = 3).

Platelet adhesion to the coated discs was clearly reduced on anti-ApoA-I antibody coated surfaces, particularly at a high density, as compared to the ApoB and isotype control (*p*<0.05; Fig 3A). Similarly, the ApoB antibody coated surface at high density showed reduced platelet adhesion compared to the isotype control (*p*<0.05). To further elucidate the implications of this latter finding we studied the influence of the oxidation state of these lipoproteins, in particular oxidized LDL, on the morphology and activation of the adhered platelets using scanning electron microscopy (Fig 3B). Only in the presence of oxidized LDL we noted significantly increased adhered platelet activation.

### In vivo study

All rabbits (n = 15; weight 3.3kg ± 0.2 kg) were successfully stented, no peri-procedural complications occurred. Two rabbits were euthanized prematurely because of gastrointestinal discomfort and significant (>10%) weight loss at day 11 and day 19 after stenting. At 28 days, the stented iliac arteries of all remaining rabbits were patent.

#### Morphometric analysis and scanning electron microscopy analysis

**Fig 4:**
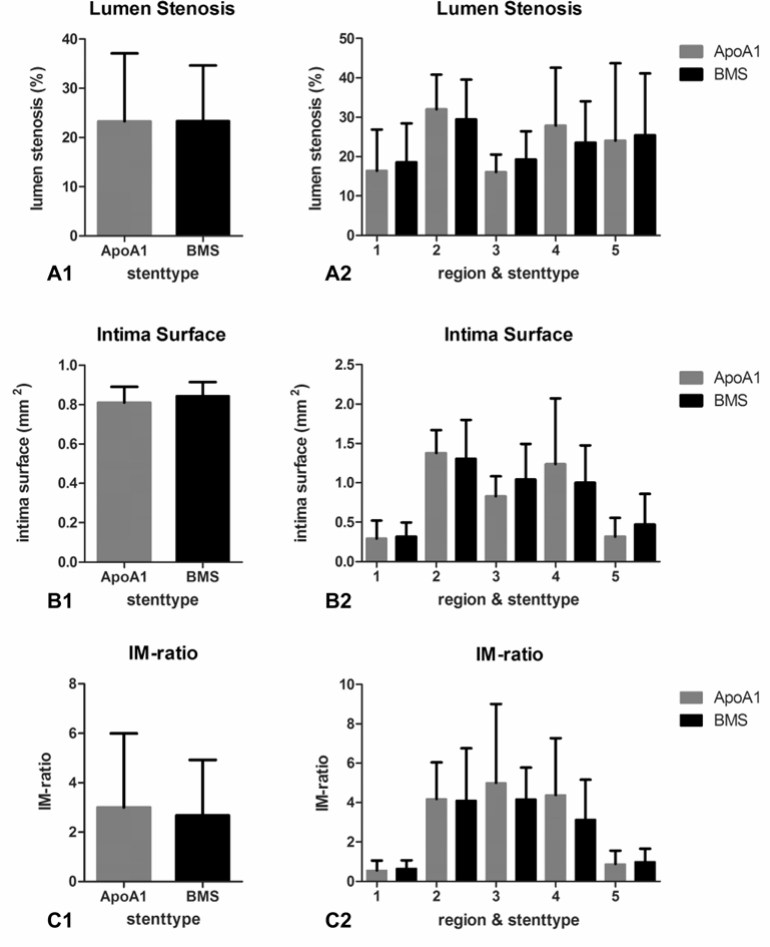
Morphometric analysis results. Lumen stenosis (A1 and A2), intima surface (B1 and B2) and IM-ratio (C1 and C2) of anti ApoA-I coated stent and BMS stent 28 days after implantation. Bars indicate mean value per section with error bars indicating the standard deviation (SD). Three left panels (A1, B1 and C1) show results of five stent regions together. No significant differences were observed between the two stent types. Three right panels (A2, B2 and C2) show results of individual stent regions in the two stent types. No significant differences were observed between the corresponding regions in the two stent types.

The Lawson-stained sections were assessed for mean lumen stenosis, intima surface and IM-ratio. As shown in panel A1 of Fig 4, anti-ApoA-I coated stents with a mean lumen stenosis of 23.3% showed stenosis rates similar to the BMS stent (*P* = 0.77). Comparison of the corresponding regions between the two stent types indicated no significant difference (panel A2). This degree of in-stent stenosis is comparable to the magnitude of stenosis observed by other groups using a similar rabbit model [28–30]. In addition, mean intima surfaces in ApoA-I coated and bare metal stents were 0.81 and 0.84 mm^2^ respectively (*P* = 0.85; panel B1). Consistent with these findings, mean IM-ratios in both stents (3.0 vs. 2.7 for ApoA-I vs. BMS, *P* = 0.28) were not different (panel C1). With respect to intima surface and IM-ratio, corresponding regions were comparable between the two stent types (panel B2 and C2).

**Fig 5:**
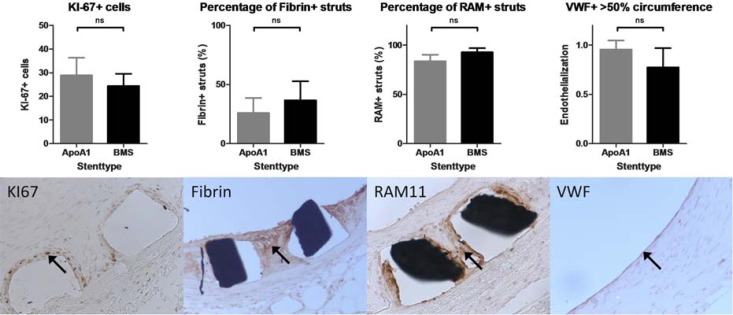
Immunohistochemical analysis. Results of immunohistochemical analysis (top) with corresponding representative examples of the immunohistochemical staining (20x objective). KI-67 (first panels), Fibrin (second panel), RAM11 (third panel) and VWF (fourth panel) are shown. Bars indicate mean score or count with error bars indicating the standard deviation (SD). Proliferation (KI-67), fibrin deposition, macrophage infiltration (RAM11) and endothelialization (VWF) were not significantly different between the two stent types.

**Fig 6:**
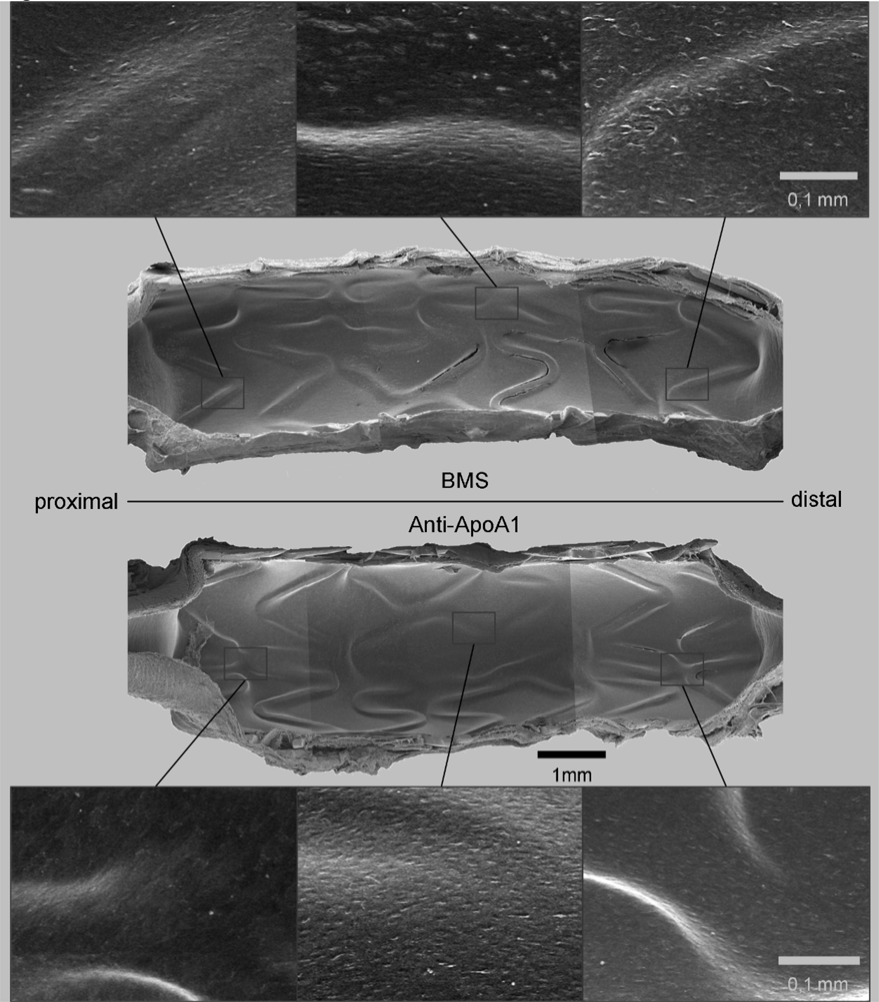
Scanning electron microscopy overview. SEM images of a 28-day BMS (upper half) and anti- ApoA-I coated stent (lower half) implanted in the rabbit common iliac artery. High magnification details of stent strut coverage are shown of three randomly chosen spots, next to the low magnification overview in the centre. Complete endothelial lining of the lumen is shown in all high-magnification details, with similar endothelial cell aspect.

We did observe a trend towards decreased RAM-11 positive regions in the vicinity of struts with the anti-ApoA-I coated stent (Fig 5; *p* = 0.056). The number of KI-67-positive (proliferating) cells did not differ between the two stents (*P* = 0.673). No difference was observed in fibrin deposition between the two stent types (*P* = 0.187). Next to the Von Willebrand staining reflecting endothelialization, the *en face* analysis of endothelium using SEM (Fig 6) shows that in both stent types, endothelialization pattern is comparable. Immunohistochemical analysis of Von Willebrand stained slides showed a trend to an increased grade of endothelialization in the ApoA-I coated stents compared to BMS (*P* = 0.072).

## Discussion

The present study shows that an anti-ApoA-I antibody coated surface, saturated with HDL, improves endothelial cell adhesion and proliferation with a concomitant decrease in thrombin generation and platelet adhesion *in vitro*. These beneficial features *in vitro* did, however, not translate into improved stent performance *in vivo* in a rabbit model of iliac artery balloon injury. The discrepancy between the *in vitro* and *in vivo* effect of anti-ApoA-I antibody coating may reflect insufficient availability of ApoA1 near the stent struts or limited capacity of HDL to attenuate the vascular injury response in an injury model of intimal hyperplasia.

### In vitro experiments

The improved endothelial cell adhesion and proliferation in our *in vitro* experiments using anti-ApoA-I coated metal discs endorse the results from earlier experimental studies on the protective functions of HDL. HDL has been shown to exert a protective effect on the endothelium by preventing apoptosis and promoting migration of endothelial cells in *in vitro* models.[31–34] In humans, HDL has consistently been shown to exert a beneficial effect on abnormal vascular reactivity.[35,36] The concomitant antithrombotic effects of ApoA-I in our *in vitro* experiments correspond to previous studies reporting antithrombotic effects of HDL. Thus, Fleisher *et al* showed that human HDL stimulates endothelial cell prostacyclin synthesis *in vitro*, [37] whereas ApoA-I-Milano resulted in decreased thrombus formation in a rat model.[10] In humans, low HDL has also been recognized as a risk factor for venous thrombotic embolism.[38,39] Mechanistically, HDL has been shown to serve as a carrier of a wide array of proteins affecting the innate immune system and proteolytic cascades.[40] In line, systemic infusion of HDL has been shown to reduce vessel wall inflammation.[41,41,42] Collectively, our *in vitro* results lend further support to the concept that the presence of ApoA-I on the stent struts may contribute to a better stent performance *in vivo*.

### Rabbit experiments

Anti ApoA-I-coated stents were not capable of decreasing the vascular response in a rabbit model of iliac balloon artery injury. Similar degree of stenosis, cell proliferation and endothelialisation were observed between ApoA-I antibody coated stents and the BMS. The apparent discrepancy between the beneficial effects *in vitro* and the absence of a beneficial effect *in vivo* may have several explanations.

First, since the stent is fenestrated, the majority of the arterial wall area is not covered by the stent struts. In contrast to coatings such as sirolimus and paclitaxel that are delivered into the local environment, the ApoA-I/HDL complex is tightly bound at the surface of the stent strut. The spatial distance between the HDL particles in relation to the lesion area between the struts may have undermined a potentially beneficial effect. In support, there was a trend towards a decreased number of inflammatory cells in the anti ApoA-I-coated stents, as well as a trend to a higher degree of endothelialisation.

Second, increased oxidative modification of fixated HDL combined with overgrowth of stent struts by intimal hyperplasia may reduce the bio-availability and potentially beneficial effects of HDL.[43] The latter may even imply that the struts become devoid of HDL delivered from the blood. Attempts to visualize the presence of HDL on stent struts using immunohistochemistry, however, failed due to the plastic embedding of the tissue.[44]

### Study limitations

Despite that inflammatory, proliferative and thrombotic stimuli within the first two weeks after stent implantation are immense, [43] the stent harvest after 28 days may have been too early to detect clinically significant differences between the two stents if in-stent restenosis occurs after longer periods of observation. Therefore, longer observation periods may yet reveal superior performance of the ApoA-I coated stent. Another drawback of the study involves the lack of information regarding the antibody binding-place availability and its effects on circulating HDL recruitment within the stented arterial wall. (S1 Fig)

### Clinical implications

Relevance of HDL mediated protection beyond reverse cholesterol transport has been widely acknowledged, whereas its impact on cardiovascular outcome remains to be proven. Positive functional effects are confirmed by our *in vitro* studies with anti ApoA-I antibody coated metallic surfaces. The attempt to translate these results to a (pre-) clinical setting has, however, failed. Whereas strategies aimed at increasing local ApoA-I concentration may still prove to be beneficial for long-term stent patency, we were unable to provide *in vivo* support for the use of anti-ApoA-I antibody coated stents to reduce intimal hyperplasia.

## Supporting Information

S1 Fig Overview of hypothesis. Schematic overview of the predefined stent regions (lower left) and a transversal section of a stented artery (upper left) The details (A-C) of an anti ApoA-I antibody coated strut compared with the strut of a bare metal stent (BMS; D-F) provide an overview of the hypothesis: anti ApoA-I antibody coated stents are implanted in the pre-injured artery (A). The coated struts attract HDL cholesterol by binding ApoA-I (B). The presence of ApoA-I and HDL prevent restenosis and promotes restoration of the endothelial layer (C). Restenosis occurs in bare metal stents (BMS), which after implantation in the artery (D) does specifically attract ApoA-I and HDL (E) and becomes overgrown by proliferating vascular smooth muscle cells, effectuating stenosis.
(TIF)

S1 Text Thrombin generation test. (DOC)

S2 Text Rabbit stent implantation model. (DOC)

S3 Text Histological and immunohistochemical staining. (DOC)

S4 Text Morphometric analysis. (DOC)

S5 Text Immunohistochemistry and the scoring systems. (DOC)

S6 Text Scanning electron microscopy. (DOC)
