# Supplementary figures and images for: Effect of Anti-ApoA-I Antibody-Coating of Stents on Neointima Formation in a Rabbit Balloon-Injury Model
Source: PLoS One. 2015 Mar 30;10(3):e0122836. doi: 10.1371/journal.pone.0122836 (PMC4378909; doi:10.1371/journal.pone.0122836)

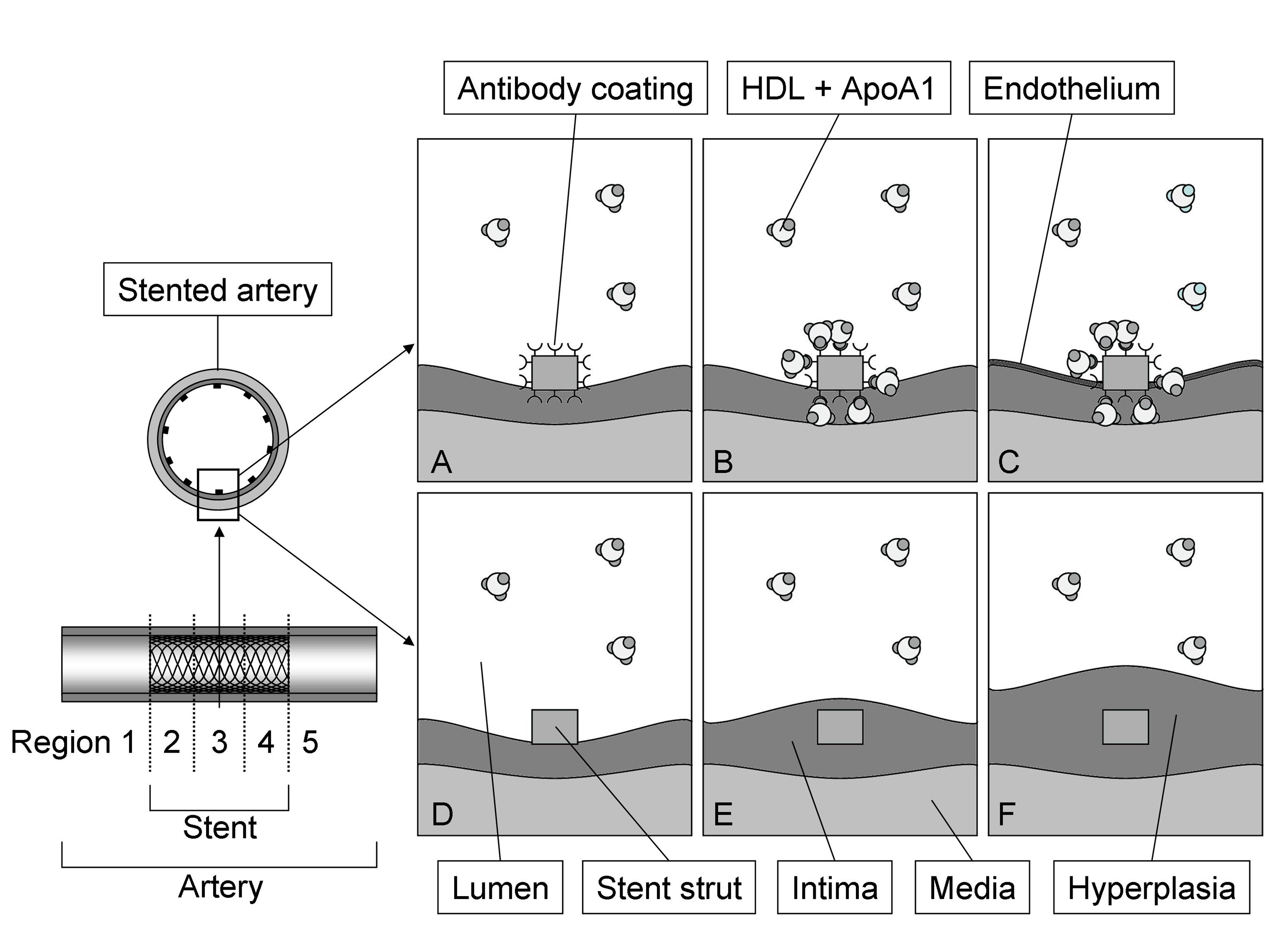

Supplement: S1 Fig — Schematic overview of the predefined stent regions (lower left) and a transversal section of a stented artery (upper left) The details (A-C) of an anti ApoA-I antibody coated strut compared with the strut of a bare metal stent (BMS; D-F) provide an overview of the hypothesis: anti ApoA-I antibody coated stents are implanted in the pre-injured artery (A). The coated struts attract HDL cholesterol by binding ApoA-I (B). The presence of ApoA-I and HDL prevent restenosis and promotes restoration of the endothelial layer (C). Restenosis occurs in bare metal stents (BMS), which after implantation in the artery (D) does specifically attract ApoA-I and HDL (E) and becomes overgrown by proliferating vascular smooth muscle cells, effectuating stenosis. (TIF) [file pone.0122836.s001.tif]
