# Supplementary material for: Effect of Anti-ApoA-I Antibody-Coating of Stents on Neointima Formation in a Rabbit Balloon-Injury Model
Source: PLoS One. 2015 Mar 30;10(3):e0122836. doi: 10.1371/journal.pone.0122836 (PMC4378909; doi:10.1371/journal.pone.0122836)
Supplement: S1 Text — (DOC) [file pone.0122836.s002.doc]

**Thrombin generation test**

Blood was collected from healthy donors in vacuum tubes containing 3.2 % sodium citrate (Greiner Bio-one, Alphen a/d Rijn, The Netherlands). Platelet-rich-plasma (PRP) was prepared by centrifugation for 15 minutes at 180g, and kept at 37 ºC. The different surfaces were pre-incubated with HDL or LDL. Discs were placed in a 96-well plate and 0.2 ml recalcified PRP, containing 400 μM fluorogenic substrate and 20 mM CaCl_2_ (final concentration) was added. This fluorogenic thrombin specific substrate, Z-Gly-Gly-Arg-AMC (Bachem Ag., Bubendorf, Switzerland), allows for the determination of thrombin generation by measuring the production of fluorescent product aminomethylcumarin (excitation 368 nm, emission 460 nm). The reaction was performed at 37 ºC for 90 minutes. The concentration of thrombin is directly proportional to the amount of fluorescent product formed over time. The lag time, also called thrombin-generation time, is appointed as the time until 2 nM thrombin was formed in the PRP. Peak height and time to peak are also extracted from curves.
