# Supplementary material for: Effect of Anti-ApoA-I Antibody-Coating of Stents on Neointima Formation in a Rabbit Balloon-Injury Model
Source: PLoS One. 2015 Mar 30;10(3):e0122836. doi: 10.1371/journal.pone.0122836 (PMC4378909; doi:10.1371/journal.pone.0122836)
Supplement: S2 Text — (DOC) [file pone.0122836.s003.doc]

**Rabbit stent implantation model**

The rabbits were anesthetized using a cocktail of Ketamine 35mg/kg and Xylazine 5mg/kg, followed by isoflurane gas anaesthesia. Blood was withdrawn and heparinised in heparin gas tubes (BD Vacutainer, BD, Playmouth, UK). After intravenous administration of heparin 100IU/kg, the left common carotid artery was cannulated with a 5F sheath introducer (Avanti +, Cordis , Fremont, USA). Under fluoroscopic guidance (Hexabrix 300; Philips BV, Eindhoven, The Netherlands) bilateral iliac artery injury was performed using an inflated 3.0 x 10mm balloon catheter (Orbus Neich, Hoevelaken, The Netherlands; 1.2:1.0 diameter ratio compared with normal vessel, mostly achieved at 10 atm) which was pulled through the external iliac artery (EIA) and deflated before removal. Subsequently, a BMS or anti- ApoA-I-coated stent was randomly placed at either side, by inflating the delivery balloon 1mm distal to the internal iliac branch, which is held for 10 seconds at a pressure of 9 atm. Stent position and patency was checked using fluoroscopy.
