# Supplementary material for: Effect of Anti-ApoA-I Antibody-Coating of Stents on Neointima Formation in a Rabbit Balloon-Injury Model
Source: PLoS One. 2015 Mar 30;10(3):e0122836. doi: 10.1371/journal.pone.0122836 (PMC4378909; doi:10.1371/journal.pone.0122836)
Supplement: S3 Text — (DOC) [file pone.0122836.s004.doc]

**Histological and immunohistochemical staining**

Sections were deplastified for 10 minutes in 100% acetone and rehydrated in series of ethanol. Sections were stained in Lawson (Klinipath, Duiven, The Netherlands) solution in order to selectively stain elastin for 60 minutes and differentiated in 100% ethanol. Sections were rinsed in tap water whereupon they were dehydrated and covered with coverslips and Pertex (Leica, Rijswijk, The Netherlands). Similarly, Haematoxylin-eosin (HE) staining (Klinipath, Duiven, The Netherlands) on one section from each region was performed to examine cell organisation and density. HE staining was also used to assess nucleus number in specific regions for immunohistochemical analyses. Of all stented arteries, a section from the second and fourth region was used for immunohistochemical staining. Semi quantitative analysis was performed on these sections immunostained with an α-smooth muscle actin (α-SMA) specific monoclonal antibody (Dako, Heverlee, Belgium; clone 1A4, dilution 1:500), to determine differences in proliferation of smooth muscle cells. Complementarily, a monoclonal antibody against Ki-67 (Dako, Heverlee, Belgium; dilution1:50) was used to score cell proliferation. For scoring inflammation grade, the rabbit macrophage-specific monoclonal antibody (RAM11, Dako, Heverlee, Belgium; dilution 1:50) was used, together with a fibrin specific antibody (America Diagnostica, Lexington, USA; dilution 1:50) to perform a fibrin deposition score. Deposited fibrin is a substrate for neutrophil adhesion in a damaged vessel wall. A fifth, monoclonal antibody against von Willebrand Factor (Abcam, Cambridge, UK; dilution 1:30) was used on sections of the stented arteries to stain endothelial cells, facilitating the ability of scoring endothelial coverage of the vessel’s lumen. All primary antibodies were detected using an horseradish peroxidise (HRP) conjugated secondary antibody (Southern Biotech, Birmingham, USA; dilution 1:100) which was visualized with 3,3'-diaminobenzidine (DAB; Sigma-Aldrich Chemie B.V., Zwijndrecht, The Netherlands).
