# Supplementary material for: Effect of Anti-ApoA-I Antibody-Coating of Stents on Neointima Formation in a Rabbit Balloon-Injury Model
Source: PLoS One. 2015 Mar 30;10(3):e0122836. doi: 10.1371/journal.pone.0122836 (PMC4378909; doi:10.1371/journal.pone.0122836)
Supplement: S4 Text — (DOC) [file pone.0122836.s005.doc]

**Morphometric analysis**

Lawson stained sections were used for morphometric analysis using a light microscope (Axiophot, Zeiss, Sliedrecht, The Netherlands) with a 1.25x or 2.5x objective and histology quantification software (QWin Leica, Rijswijk, The Netherlands). Picture colours were converted to 8-bit grey values, with the use of a saturation threshold, outer elastic lamina (OEL), inner elastic lamina (IEL) and lumen outline were recognized. Stent struts and surrounding shrink-artefacts were outlined and summed and called stent strut surface. Lumen surface was measured as content of lumen outline. Intima surface was calculated by deducting lumen surface and summed strut surface from surface inside IEL. Media surface was calculated by deducting the surface inside IEL from the surface inside OEL. Intima-media (IM-) ratio was calculated by dividing intima surface by media surface. Lumen stenosis was calculated by taking the proportion of surface within IEL which was occupied by intima and strut surface with respect to total IEL surface. Maximal intima surface was measured by arbitrarily measuring the tunica intima at its thickest part viewed from the centre of the lumen. Mean surfaces of three sections within a region were calculated.
