# Supplementary material for: Effect of Anti-ApoA-I Antibody-Coating of Stents on Neointima Formation in a Rabbit Balloon-Injury Model
Source: PLoS One. 2015 Mar 30;10(3):e0122836. doi: 10.1371/journal.pone.0122836 (PMC4378909; doi:10.1371/journal.pone.0122836)
Supplement: S5 Text — (DOC) [file pone.0122836.s006.doc]

**Immunohistochemistry and the scoring systems**

Proliferation was scored by dividing the Ki-67 positive cells by the total number of cells in the HE stained coupes. The degree of endothelialisation was scored by determining the number of von Willebrand positive cells and was categorized as 0-50% and 50-100%.

The inflammation score was determined by dividing the number of struts that stained positive for RAM11, by the total number of struts. A fibrin deposition score was based on dividing the number of struts that stained positive for fibrin staining by the total strut count in fibrin stained sections.
