# Supplementary material for: Effect of Anti-ApoA-I Antibody-Coating of Stents on Neointima Formation in a Rabbit Balloon-Injury Model
Source: PLoS One. 2015 Mar 30;10(3):e0122836. doi: 10.1371/journal.pone.0122836 (PMC4378909; doi:10.1371/journal.pone.0122836)
Supplement: S6 Text — (DOC) [file pone.0122836.s007.doc]

**Scanning electron microscopy**

To further evaluate stent endothelialisation, stents (n=1 per condition) were cut in a sagittal fashion and incubated with 2% aqueous osmium tetroxide for 45 min, dehydrated in a graded ethanol series and then critical point dried in liquid CO_2_ using the Balzers CPD-010 (Balzers Instruments, Liechtenstein). Stent halves were mounted on aluminium stubs (Ted Pella Inc, Redding, USA) and sputter coated with gold/palladium using the Polaron E5100 SEM coating system (Thermo VG Scientific, Waltham, USA). Endothelial facing layer was examined on a scanning electron microscope at 15kV and at magnifications ranging from 400 to 4000x.
